# Epsilon‐Negative Derived Electromagnetic Wave Absorption of Magnetic Metacomposites by Bifunctional Phase Paradigm

**DOI:** 10.1002/advs.202516616

**Published:** 2025-11-18

**Authors:** Xiance Zhu, Yunpeng Qu, Qiuyun Yang, Jing Mao, Xiaosi Qi, Yunlei Zhou, Chunyuan Deng, Shicheng Qiu, Yao Liu

**Affiliations:** ^1^ College of Physics Guizhou University Guiyang 550025 China; ^2^ Hangzhou Institute of Technology Xidian University Hangzhou 311231 China; ^3^ School of Electronic Engineering and Automation Guilin University of Electronic Technology Guilin 541004 China; ^4^ Department of Electronic & Computer Engineering Hong Kong University of Science and Technology Hong Kong 999077 China; ^5^ Key Laboratory for Liquid‐Solid Structural Evolution and Processing of Materials (Ministry of Education) Shandong University Jinan 250061 China

**Keywords:** electromagnetic wave absorption, metacomposites, negative permittivity, percolation

## Abstract

Electromagnetic (EM) metacomposites possess exceptional capabilities for manipulating EM waves within the microwave frequency spectrum. However, their current designs lack a systematic structural framework, leading to ambiguity in the regulation of their EM parameters. This study presents a multifunctional phase paradigm for metacomposites that combines the conductive phase of graphene (GR) with the insulating phase of Fe_3_O_4_. In this arrangement, Bi nanoparticles act as electronic transport bridges, effectively linking GR and Fe_3_O_4_ particles. This structure‐function relationship facilitates the achievement of a *ε'*‐negative response in the frequency range of 10^4^–10^5^ across the 10 MHz –1 GHz band, demonstrating stable regulation. The Drude model is employed to elucidate the frequency dependence of negative permittivity, highlighting the plasmonic oscillation effect within GR networks. Meanwhile, analysis of EM simulation results indicates that the *ε'*‐negative medium induces significant EM wave loss due to enhanced polarization effects stemming from the carbon network and the multi‐interface structure of the GR/Bi@Fe_3_O_4_ metacomposites. The optimized *ε'*‐negative metacomposites achieve a reflection loss of −50.69 dB at 13.6 GHz, driven by the synergistic effects of GR's conductive loss, Fe_3_O_4_’s magnetic loss, and multi‐scale interface polarization effects.

## Graphical abstract

An epsilon‐negative response of up to 100000 in the radio frequency band is achieved by creating a metacomposite system with graphene/iron (III) oxide as the main body. The magnetic metacomposites are just like the mythical creature Qiongqi in Chinese mythology, capable of swallowing electromagnetic wave pollution that falls from the sky.

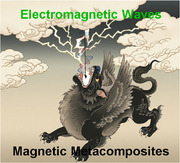

## 1 Introduction

Since the early 21st century, electromagnetic (EM) metamaterials have led to significant scientific advancements, particularly through the pioneering work of Pendry and Smith, who introduced innovative structural designs involving periodic split‐ring resonators and metal wire arrays.^[^ ^1^ ^]^ A landmark achievement was the first successful observation of a negative refractive index within the microwave frequency range (4.7–5.5 GHz). This was accomplished through the simultaneous realization of negative dielectric permittivity (*ε'* < 0) and negative magnetic permeability (*µ'* < 0).^[^ ^2^ ^]^ This groundbreaking research overcame the theoretical constraints of conventional materials regarding EM parameters, paving the way for the development of extraordinary EM devices such as radar systems, infrared stealth technologies, and perfect absorption antenna covers, meta‐lenses, etc.^[^ ^3^ ^]^

Due to variations in structural design paradigms, EM response characteristics, and material systems, EM metamaterials have evolved into distinct branches, including metasurfaces with 2D structures,^[^ ^4^ ^]^ *ε'*‐near‐zero (ENZ) metamaterials, and metacomposites.^[^ ^5^ ^]^ Among these categories, metacomposites have gained prominence for achieving negative EM parameters specifically in the radio frequency band (kHz‐GHz). This success is largely attributed to their ability to leverage the intrinsic physical properties of natural materials while offering high design flexibility, a diverse range of material options, and adjustable performance characteristics inherent in random composite structures. For instance, researchers have demonstrated the achievement of negative parameters in the MHz frequency band by incorporating functional phases of metals like Fe, Ni, and Cu within a ceramic matrix, thus creating a randomly constructed 3D network structure that exploits their plasmonic oscillation effect.^[^ ^6^ ^]^ The resultant negative EM response is marked by high intensity, broad bandwidth, and significant loss.

Nevertheless, in most application scenarios, the need for lightweight, integrated, and miniaturized solutions poses considerable challenges for metacomposite material systems that depend on metals as functional phases. To overcome these hurdles, researchers have investigated strategies such as metal nanosizing, exemplified by the development of metacomposites primarily composed of silver nanowires, aimed at enhancing the controllability of the composite structure.^[^ ^7^ ^]^ Recent studies indicate that employing carbon nanomaterials as an alternative may provide a more effective solution. Carbon nanomaterials, which include graphene (GR), carbon nanotubes (CNT), carbon black, carbon aerogels, and amorphous carbon, offer a variety of geometric configurations, advantageous physical properties, and chemical stability, thereby presenting significant benefits for composite construction.^[^ ^8^ ^]^

There is no doubt that the carbon network plays a crucial role in achieving negative parameters in metacomposites. By modulating this network, the intensity and range of negative parameters can be effectively controlled. For example, incorporating Ni and MnFe_2_O_4_ into CNT can balance the effective carrier concentration within the metacomposites, leading to wideband ENZ and negative permittivity. Additionally, carbon aerogel and an amorphous carbon network can be carbonized under varying temperature conditions, allowing for the reconstruction of their porous structure and degree of graphitization, which facilitates the regulation of the *ε'*‐negative magnitude.^[^ ^9^, ^10^ ^]^ Moreover, metacomposites have shown promising applications in areas such as microwave absorption. Their tunable EM parameters and flexible structural design offer new opportunities for developing high‐performance absorptive materials. However, the importance of the “matrix phase” in constructing metacomposites is often overlooked, which significantly limits their applicability in fields such as EM wave shielding, absorption, meta‐capacitors, and sensing. Specifically, past studies have shown that insulating phases, such as ceramics and polymers, used to support carbon networks primarily served as structural components. This has resulted in the negative EM response, which is solely attributed to the carbon networks, being inadequate for meeting the demands of multifunctional applications once realized in metacomposites.^[^ ^11^ ^]^

To overcome this limitation, we propose a bifunctional phase metacomposite based on GR/Bi@Fe_3_O_4_, which achieves a negative permittivity response through a 3D conductive GR network while also providing magnetic properties through insulating Fe_3_O_4_. This metacomposite successfully achieves a controllable *ε'*‐negative response in the 10 MHz–1 GHz frequency band, with maximum response magnitudes between 10000 and 100000. Meanwhile, due to its superior magnetic properties, the metacomposite exhibits a maximum reflection loss of −50.69 dB at 13.6 GHz.

Moreover, we systematically examined the structure‐activity relationship and impedance characteristics of the composites, utilizing EM simulation technology and thermal infrared characterization to investigate their EM loss mechanisms. The proposed bifunctional metacomposites are expected to establish a new paradigm for structural design, elucidating the regulatory mechanisms of *ε'*‐negative response while enhancing multifunctional applications in areas such as EM wave shielding and absorption.

## 2 Results and Discussion

### 2.1 Structural Characteristics of Metacomposites

**Figure 1:**
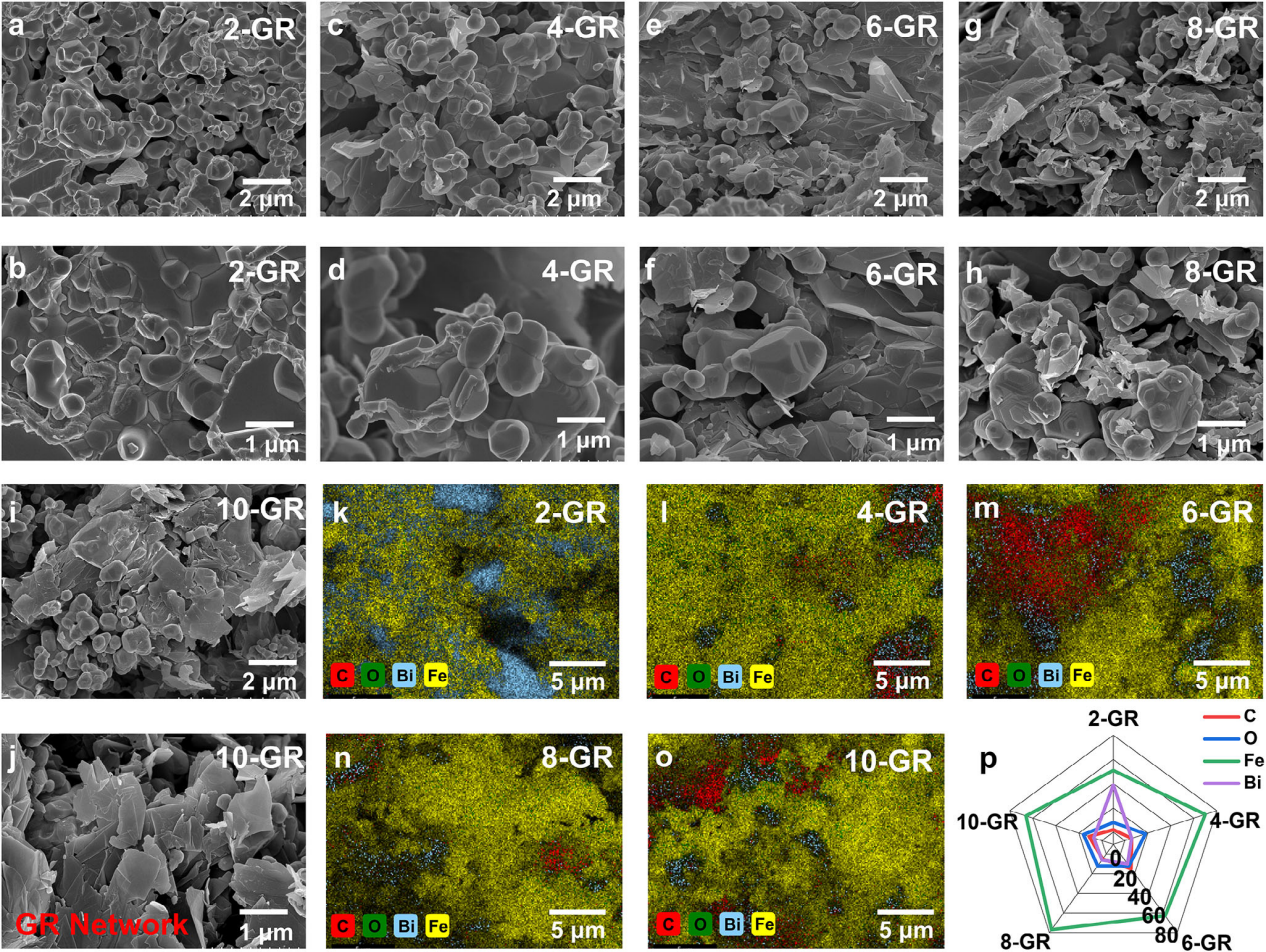
SEM images a–j), EDS maps k–o) and elemental contents p) of GR/Bi@Fe_3_O_4_ composites with different mass ratios of GR fillers.

As shown in **Figure** **1**, Bi and Fe_3_O_4_ particles possess an irregular block‐like morphology with a nonuniform size distribution, while GR exhibits its characteristic single‐layer, 2D sheet‐like structure. As the concentration of GR increases, the GR sheets progressively coat the surfaces of the Bi and Fe_3_O_4_ particles. This transition in distribution morphology evolves from initially isolated sheets to a continuous 3D conductive network.^[^ ^12^ ^]^ Specifically, when the GR doping concentration reaches 10 wt.%, a significant and continuous GR 3D network structure is formed in the sample. Further analysis using EDS reveals the distribution of four elements—C, O, Bi, and Fe—within the composite. The EDS elemental distribution analysis clearly illustrates the spatial arrangement of these elements, with variations in their content exhibiting distinct distribution patterns.^[^ ^13^ ^]^

**Figure 2:**
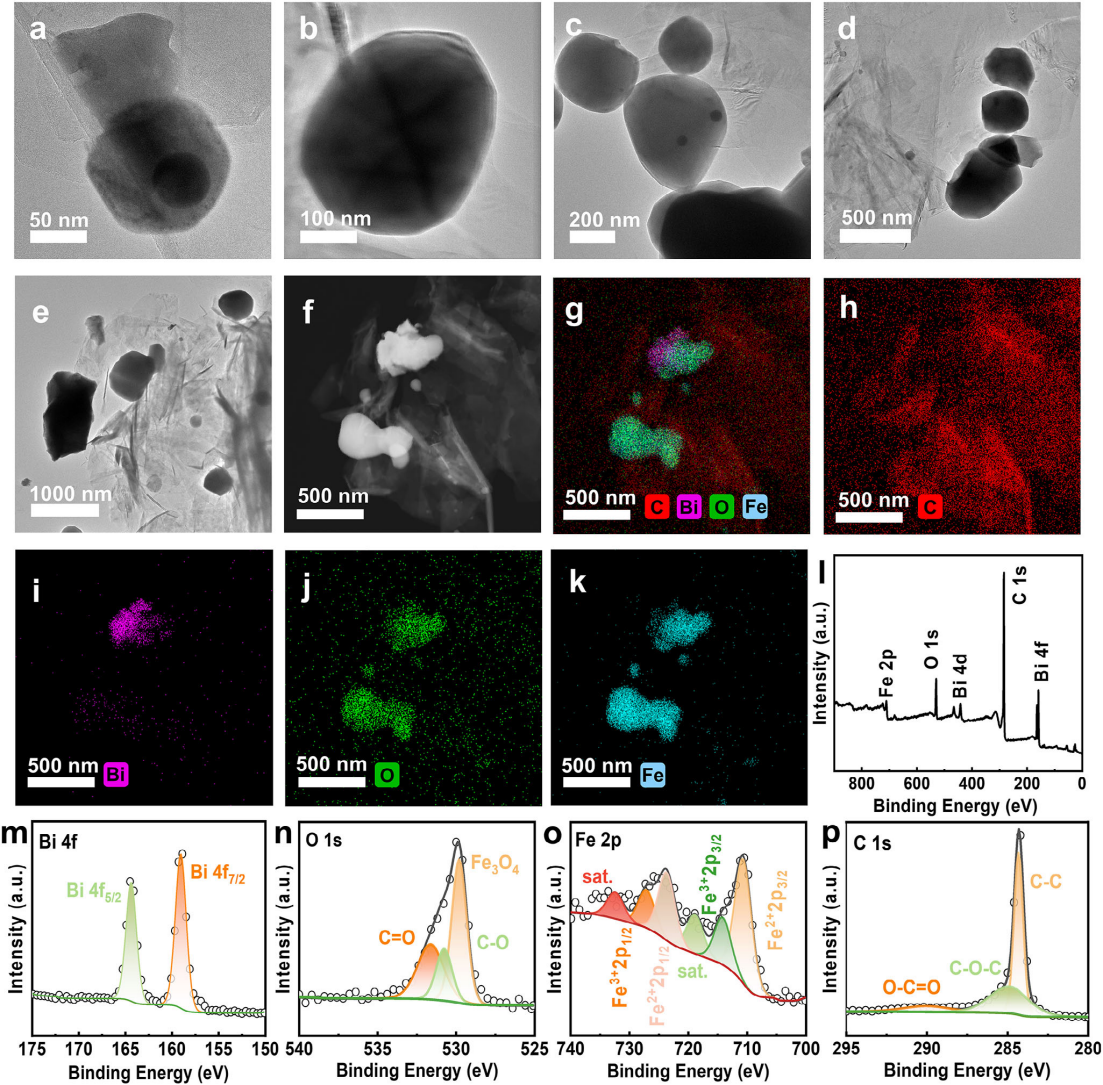
TEM images a–e), EDS mapping images f–k), and XPS images l–p) of the 10‐GR sample.

As shown in **Figure** **2**a–e, GR displays a distinct single‐layer, 2D plate‐like morphology. In this structure, larger block‐like particles represent Fe_3_O_4_, while smaller particles of Bi are dispersed around the Fe_3_O_4_. EDS analysis (Figure 2f–k) provides a more intuitive observation: C (representing GR) is distributed most widely and uniformly, consistent with its large‐sized 2D plate‐like structure; the distribution regions of Fe and O elements highly overlap, confirming that they form a compound; while Bi elements are primarily distributed in the peripheral regions of Fe‐O compound particles.^[^ ^14^ ^]^ XPS analysis results (Figure 2l–p) reveal distinct peaks for C, O, Bi, and Fe, with no additional elemental peaks detected, confirming that the material is a composite made up of these four elements. Importantly, the peak for O at 259 eV indicates a metal‐oxygen bond, and based on mapping results, this is identified as an Fe‐O compound. Furthermore, the Fe spectrum exhibits characteristic peaks indicative of both +2 and +3 oxidation states, preliminarily suggesting that the compound is Fe_3_O_4_.^[^ ^15^ ^]^ The specific phase composition requires further confirmation through subsequent XRD analysis.

**Figure 3:**
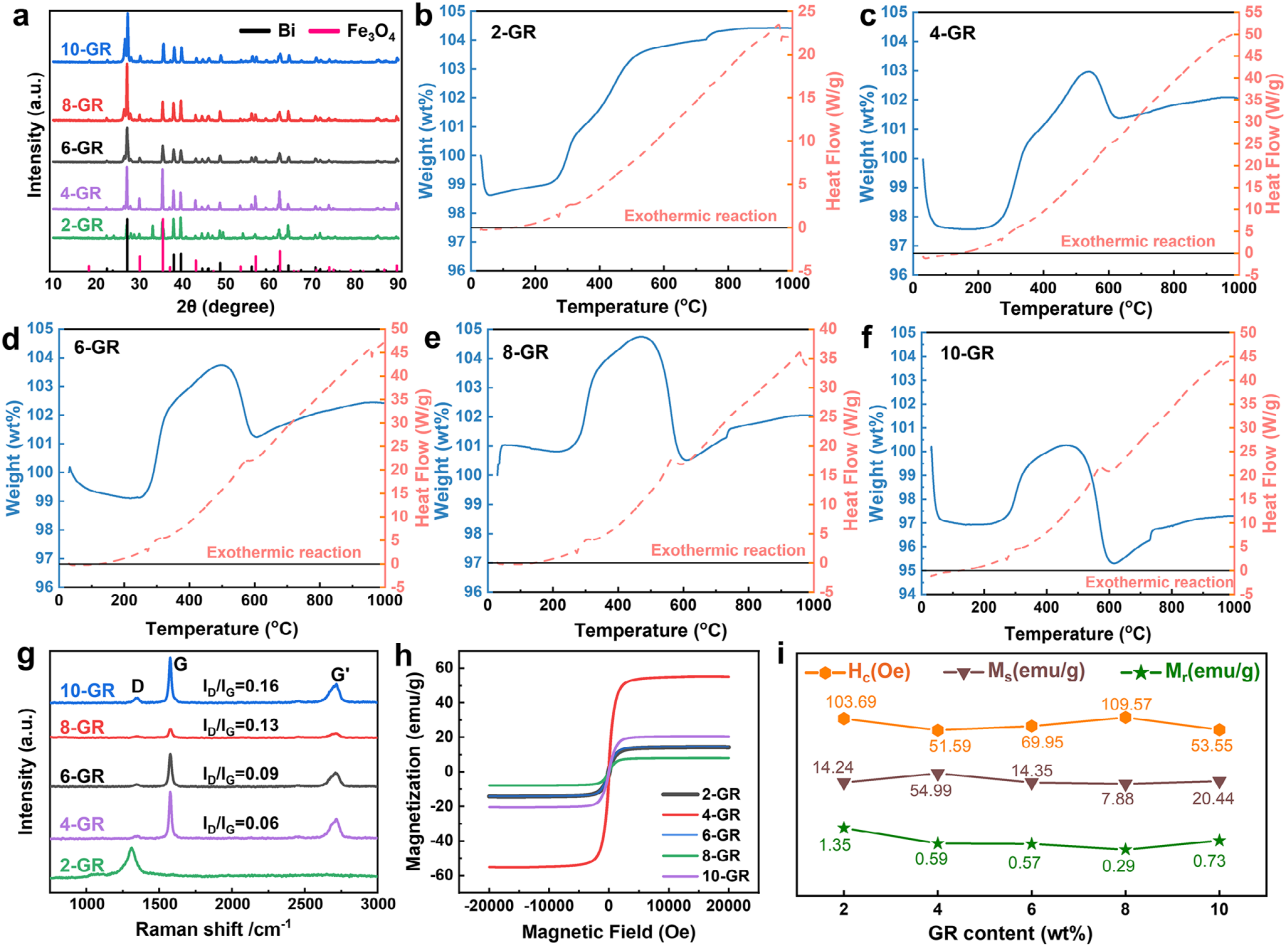
XRD a), TG‐DSC b–f), Raman g) and hysteresis loop plots h) of GR/Bi@Fe_3_O_4_ composites with different GR content. Trends of saturation magnetization, residual magnetism, and coercive force varying with GR content.

The XRD analysis results (**Figure** **3**a) reveal that most samples present characteristic diffraction peaks that align perfectly with the standards for Fe_3_O_4_ and Bi, with no impurity phases observed. This finding confirms the successful synthesis of the GR/Bi@Fe_3_O_4_ three‐phase composites. However, at a GR content of 2 wt.%, additional diffraction peaks corresponding to the Bi_2_Fe_4_O_9_ phase appear alongside the characteristic peaks of Fe_3_O_4_ and Bi (Figure S9, Supporting Information). This phenomenon can be attributed to the following: in the initial raw material without added GR, the main component was Bi_2_Fe_4_O_9_; as the GR content increased, the reducing effect of GR caused Bi to precipitate from Bi_2_Fe_4_O_9_, and the matrix material transformed into Fe_3_O_4_.^[^ ^16^ ^]^ Notably, at 2 wt.% GR, the insufficient reducing agent leads to incomplete reduction of Bi, resulting in the coexistence of Bi_2_Fe_4_O_9_, GR, Fe_3_O_4_, and Bi in the sample. In contrast, when the GR content reaches 4 wt.% or higher, diffraction peaks of Bi_2_Fe_4_O_9_ are no longer detected, indicating that Bi has been fully reduced, ultimately yielding a three‐phase composite material of GR/Bi@Fe_3_O_4_.^[^ ^17^ ^]^ This observation further supports the role of reduced Bi as a bridge connecting GR and Fe_3_O_4_.

The TG‐DSC test results (Figure 3b–f) indicate that all samples display comparable thermal behaviors when subjected to a constant heating rate in an air atmosphere. As the temperature rises, GR experiences oxidative weight loss accompanied by exothermic reactions. At ≈300°C, Bi and Fe_3_O_4_ are oxidized to Bi_2_O_3_ and Fe_2_O_3_, respectively, leading to the formation of bismuth iron oxide. Initially, this process is characterized by an endothermic weight gain, which is followed by mass loss due to the volatilization of Bi_2_O_3_, continuing until reaching 600 °C. Notably, the 2‐GR sample showed minimal mass loss, likely due to the presence of Bi_2_Fe_4_O_9_, which facilitates the formation of stable bismuth‐iron oxides between Bi_2_O_3_ and Fe_2_O_3_, thereby reducing the volatilization of Bi_2_O_3_. In the temperature range of 600–1000 °C, Bi_2_O_3_ continues to volatilize, while the secondary oxidation of Bi and Fe_3_O_4_ results in an overall endothermic characteristic for the sample, accompanied by a slight mass increase.^[^ ^18^ ^]^

Raman spectroscopy (Figure 3g) provides a systematic analysis of the graphitization degree and structural defects in the composite material containing GR. The D peak reflects the density of defects, the G peak indicates the level of graphitization, and the G' peak, or the 2D peak, signifies the second‐order overtone of the D peak, which is particularly sensitive to the number of GR layers.^[^ ^19^ ^]^ Different layer numbers result in different peak shapes, with the symmetric single‐peak feature confirming a single‐layer structure of GR. At a GR content of 2 wt.%, only the D peak is prominently observed, while both the G and G' peaks are absent. This observation suggests that at this ratio, the GR structure is significantly defective and highly disordered. As the GR content increases by 2 wt.%, the I_D_/I_G_ ratio rises incrementally by 0.03. This increase in defect density arises from both chemical and physical mechanisms: the C1s spectrum in XPS (Figure 2p) reveals the presence of C‐O‐C and O‐C═O bonds, indicating partial oxidation of GR. The formation of oxygen‐containing functional groups interrupts the sp2 conjugated domains, while the interface contact between GR and Fe_3_O_4_/Bi generates cumulative local strain and bending within the GR plane.^[^ ^20^ ^]^ Although the modest increase in I_D_/I_G_ (0.03) indicates limited oxidation, this slight oxidation reduces carrier mobility without resulting in excessively high free carrier concentrations in GR. As a result, material hydrophilicity is enhanced, and catalytic active sites are provided.^[^ ^21^ ^]^ Hysteresis loop tests (Figure 3h,i) demonstrate that the composite material not only possesses significant intrinsic magnetic susceptibility but also allows for effective tuning of its magnetic properties by adjusting the GR filler content. This offers a new level of flexibility in the design of bifunctional phase materials.^[^ ^22^ ^]^

### 2.2 Electrical Percolation of GR/Bi@Fe_3_O_4_ Metacomposites

**Figure 4:**
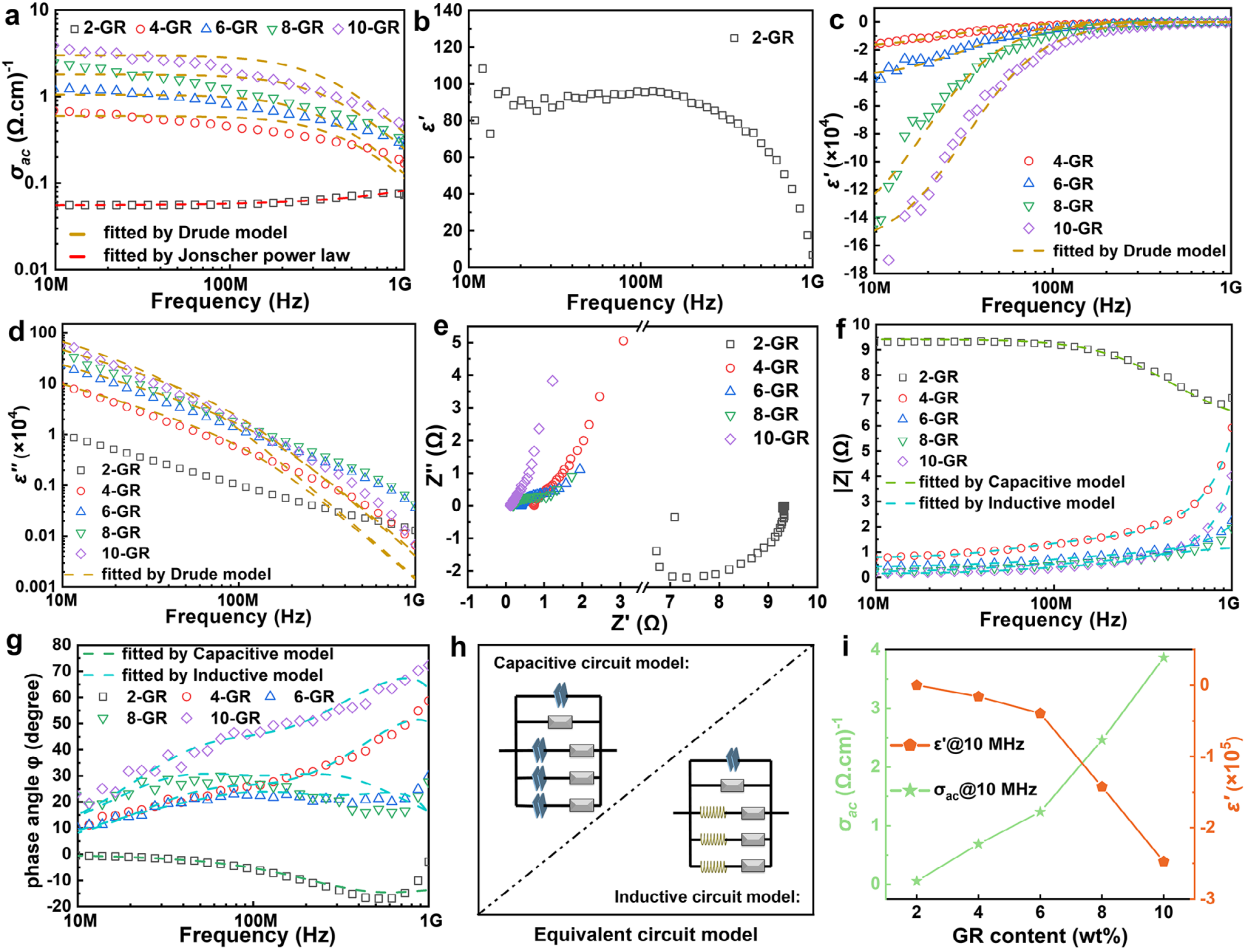
Frequency distribution of AC conductivity a), permittivity b–d), Nyquist plot e), impedance magnitude f), phase angle g), equivalent circuit model plots h), and AC conductivity and real permittivity at 10 MHz i) for GR/Bi@Fe_3_O_4_ composites with different mass ratios of GR fillers.

**Figure** **4** illustrates the results of electrical performance tests conducted on the GR/Bi@Fe_3_O_4_ composites, along with a detailed analysis. In examining the frequency dependence of AC conductivity (Figure 4a), it is observed that when the GR loading is at 2 wt.%, the AC conductivity behavior of the sample adheres closely to Jonscher's power law relationship^[^ ^23^ ^]^:

(1) $$\sigma_{ac}=\sigma_{dc}+A{\left(2\pi f\right)}^{n}$$

where *σ_ac_*, *σ_dc_*, *A*, and *n* denote the AC conductivity, DC conductivity, exponent pre‐factor, and exponent, respectively. In the low‐frequency range, *σ_ac_* is predominantly influenced by *σ_dc_*, resulting in a plateau characteristic. Conversely, in the high‐frequency range, *σ_ac_* exhibits a marked frequency‐dependent increase, indicative of the hopping conduction mechanism. As the GR loading increases, the composite material's *σ_ac_* shows a significant upward trend, attributed to the excellent conductivity of GR. Notably, the metallic properties of Bi facilitate effective electronic transport between GR and Fe_3_O_4_, significantly reducing interfacial contact resistance and enhancing the overall conductivity of the material.

Further examination reveals that *σ_ac_* gradually changes in the low‐frequency region but experiences a sharp decline as the frequency increases in the high‐frequency region. This phenomenon can be elucidated by the Drude model^[^ ^24^ ^]^:

(2) $$\sigma_{ac}=\frac{\sigma_{dc}\omega_{\tau }^{2}}{\omega^{2}+\omega_{\tau }^{2}}$$

where *ω* represents the angular frequency of the external electric field and *ω_τ_* signifies the relaxation rate. Similar to Jonscher's power law, *σ_ac_* approaches *σ_dc_* at low frequencies; however, at high frequencies (*ω* ≫ *ω_τ_*), *σ_ac_* significantly decreases. This decrease suggests that free electrons in the sample find it challenging to keep pace with the rapid fluctuations of the high‐frequency electric field, resulting in their accumulation at the material's surface and a subsequent reduction in overall conductivity, demonstrating the typical skin effect. The skin effect, a crucial aspect of metallic conductivity mechanisms, is described by the skin depth (*δ*)^[^ ^25^ ^]^:

(3) $$\delta =\sqrt{\frac{2}{\omega \sigma_{dc}\mu }}$$

where *μ* represents the static magnetic permeability. These observations indicate that at a GR loading of 4 wt.%, a well‐developed conductive network forms within the composites. On the one hand, GR transitions from its initial dispersed state into a continuous, interconnected conductive network. On the other hand, Bi nanoparticles effectively occupy the interfacial voids between GR and Fe_3_O_4_, creating 3D conductive pathways and significantly enhancing the overall electron transport efficiency of the electrode. Consequently, at 4 wt.% GR loading, the primary conductive mechanism of the composite material undergoes a fundamental shift, transitioning from electron hopping conduction to metallic conduction, with the directed migration of free electrons becoming the main contributor to conductivity.^[^ ^26^ ^]^

### 2.3 Epsilon‐Negative Response of GR/Bi@Fe_3_O_4_ Metacomposites

The analysis of dielectric properties (Figure 4b–d) systematically examines how the dielectric behavior of the composites evolves with varying GR loadings. The findings indicate that the amount of GR filler is a crucial factor in influencing the negative dielectric response. In the case of the 2‐GR sample, the permittivity remains positive throughout the entire frequency range tested, clearly signifying that the composite has not yet reached the percolation threshold, and continuous conductive pathways have not been formed within the material. This sample also demonstrates a strong frequency dependence in permittivity, which shows a rapid decline as frequency increases. This behavior can primarily be attributed to leakage currents generated by isolated GR particles when exposed to high‐frequency EM fields.^[^ ^27^ ^]^ When the GR content is increased to 4 wt.% or more, all samples exhibit negative permittivity characteristics across the testing frequency range. As the GR content increases, the negative permittivity also increases significantly; however, it gradually decreases as frequency increases. This critical phenomenon can be elucidated using the classical Drude model^[^ ^28^ ^]^:

(4) $$\varepsilon^{\prime }=1-\frac{\omega_{p}^{2}}{\omega^{2}+\gamma^{2}}$$

(5) $$\omega_{p}=\sqrt{\frac{n_{eff}e^{2}}{m_{eff}\varepsilon_{0}}}$$

where *γ* represents the dissipation constant, *ω_p_* is the plasma frequency, while *n_eff_* and *m_eff_* denote the effective electron density and mass, respectively, and *ε₀* is the vacuum permittivity. When the test frequency *ω* is below *ω_p_*, the free electron gas within the material undergoes plasma oscillations, resulting in negative permittivity. This phenomenon confirms that the sample has exceeded the percolation threshold and established an effective conductive network. Since the *ω_p_* is primarily determined by *n_eff_* and *m_eff_*, as the GR loading increases to 10 wt.%, the concentration of free electrons in the composites significantly increases, causing *ω_p_* to shift to higher frequencies and allowing for a greater negative permittivity over a wider frequency range.^[^ ^29^ ^]^

The variation in dielectric loss (*ε''*) (Figure 4d) provides insight into the evolution of the conductive network within the composites and the transformation of its loss mechanism. Below the percolation threshold (2 wt.%), *ε''* primarily originates from the carrier hopping conduction mechanism, which includes relaxation polarization and the conductance loss of localized carriers. As frequency increases, limited carrier mobility limits their ability to keep pace with the alternating electric field, resulting in a weakened polarization response that exhibits typical exponential decay characteristics.^[^ ^30^ ^]^ When the GR content reaches the percolation threshold (4 wt.% or higher), the formation of conductive pathways allows carriers to escape localized confinement, shifting the loss mechanism to one dominated by metal conduction loss of free electrons. This transition can be quantitatively described by the *ε''* equation in the Drude model^[^ ^31^ ^]^:

(6) $$\varepsilon^{\prime \prime }=\frac{\gamma \omega_{p}^{2}}{\omega \left(\omega^{2}+\gamma^{2}\right)}$$

In the low‐frequency region, the experimental data align closely with the conduction loss behavior predicted by the Drude model. However, in the high‐frequency region, despite theoretical predictions indicating that losses should decrease exponentially with increasing frequency, the actual measured values are consistently slightly higher than the theoretical fitting results. This discrepancy suggests that, alongside conduction losses, the material experiences additional loss mechanisms, such as dipole polarization and interface polarization. As the GR filling content increases, the *ε''* values show a general upward trend, attributed to the growing accumulation of charge in the interface regions of the composites, which significantly enhances the interface polarization effect.^[^ ^32^ ^]^ These observations not only clarify the formation process of the conductive network but also illustrate the evolution of loss mechanisms in composites at varying frequencies and their association with microstructure.

### 2.4 Impedance Response of GR/Bi@Fe_3_O_4_ Metacomposites

The analysis of the impedance spectrum using the ZSimWin software (Figure 4e–h) systematically reveals the impedance response characteristics of the composites for different GR fillings. In the complex impedance expression *Z = Z' + jZ''*, the change in the sign of the reactance component *Z'' = Z_L_ ‐Z_C_* directly reflects the transformation mechanism of the material's impedance characteristics. A quantitative analysis of the Nyquist plot (Figure 4e) demonstrates that at a GR content of 2 wt.%, the negative *Z''* value indicates that capacitive reactance (*Z_C_*) dominates, showcasing typical dielectric behavior. However, as the GR content rises to 4 wt.% or more, the *Z''* value transitions from negative to positive, confirming that inductive reactance (*Z_L_*) begins to dominate the material's impedance response, signifying the establishment of a conductive network.^[^ ^33^ ^]^

The frequency dependence of the impedance modulus (Figure 4f) further supports the observed transition process. For the 2‐GR sample, *|Z|* decreases steadily as frequency increases, aligning with the fundamental behavior of a capacitive material (*Z_C_ = 1/ωC*). In contrast, samples with 4 wt.% and higher show an upward trend in *|Z|* with increasing frequency, indicative of an inductive material (*Z_L_ = ωL*). Phase angle analysis (Figure 4g) offers additional clarity: the capacitive region (2 wt.%) displays a voltage phase that lags behind the current (negative phase angle), while the inductive region (4 wt.% and above) shows the current phase lagging behind the voltage (positive phase angle). Consequently, the frequency characteristics of both the impedance modulus and phase angle (Figure 4f,g) align well with the equivalent circuit model (Figure 4g).^[^ ^34^ ^]^ Importantly, the results presented in Figure 4i reveal that as the GR content increases, *σ_ac_* rises significantly while *ε'* decreases, reinforcing the influence of GR content and conductive network formation on the material's overall electrical properties. The impedance analysis of these systems not only quantitatively captures the transition from dielectric to conductive properties in the composites but also provides crucial experimental evidence for understanding microstructural changes near the conductive percolation threshold.^[^ ^35^ ^]^ By thoroughly examining *σ_ac_*, dielectric properties, and impedance characteristics, we have established a structure‐property relationship between GR loading, microstructure, and electrical properties, thereby laying a solid theoretical foundation and offering experimental guidance for the design and optimization of multifunctional GR/Bi@Fe_3_O_4_ composites.

### 2.5 EM Wave Absorption of GR/Bi@Fe_3_O_4_ Metacomposites

**Figure 5:**
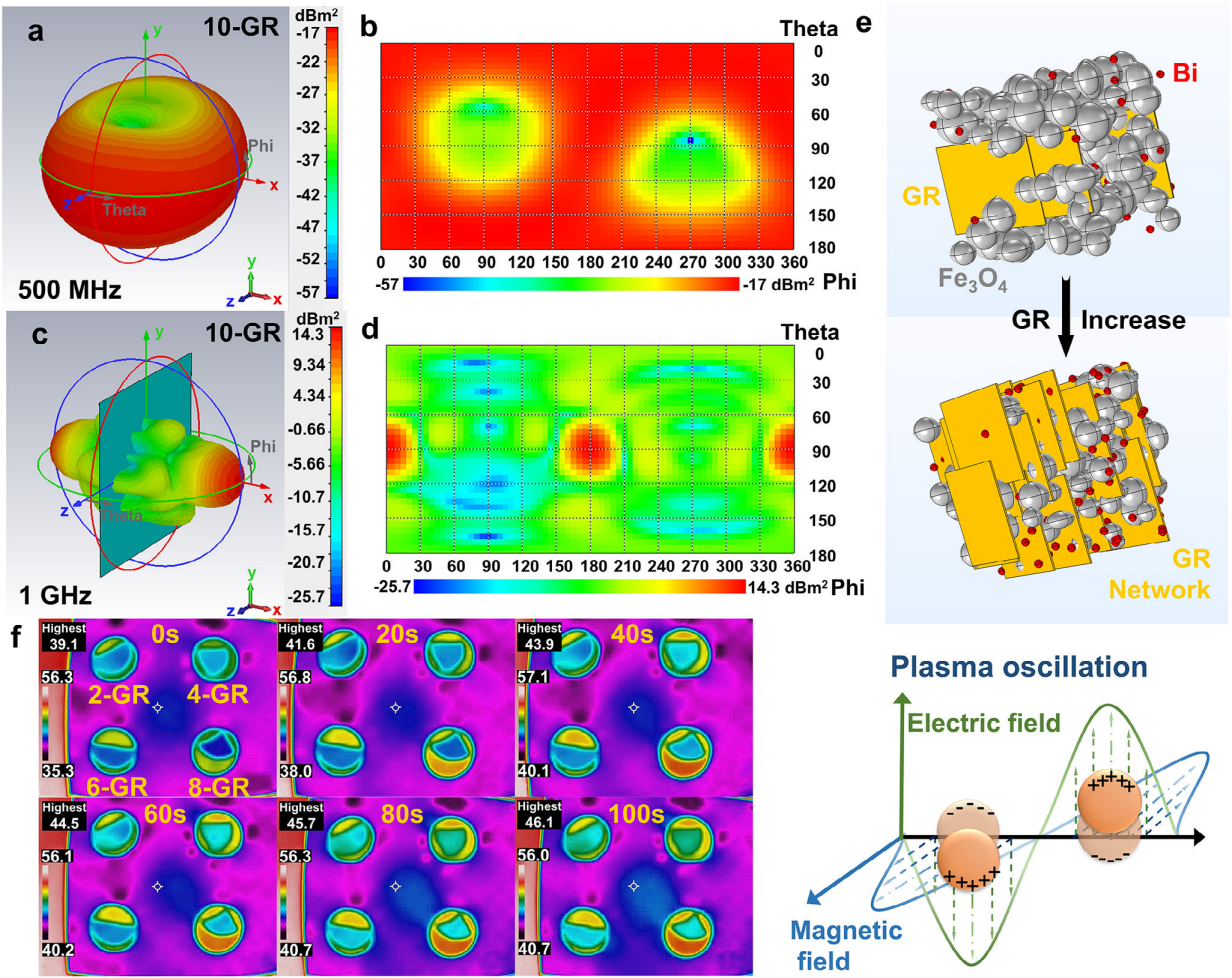
3D radar wave scattering signal at 500 MHz and 1 GHz of the 10‐GR sample a–d). Schematic illustrating the microstructural evolution and response mechanism of the metacomposites, e,f). The infrared thermal imaging diagram of the GR/Bi@Fe_3_O_4_ placed on a PLA plate and heated at 60°C for 100 g).

As illustrated in **Figure** **5**a–d, computational results from CST simulation software reveal that, at a working frequency of 500 MHz, this material system exhibits unique EM response characteristics. At this frequency, the material demonstrates a significantly negative *ε'* and an enhanced *ε''*, resulting in a negative radar cross‐section (RCS) across the entire azimuth angle range, with a minimum RCS value reaching as low as −57 dBm^2^. Compared to the test results at 1 GHz, the performance at the 500 MHz frequency band highlights a notable advantage. This effect arises from the stronger interaction between the collective oscillation of free electrons within the material and the incident EM waves under low‐frequency conditions (Figure 5e,f).^[^ ^36^ ^]^

The analysis of microstructural evolution (Figure 5e) reveals a clear transformation in the composites as the GR filling ratio increases, shifting from isolated, dispersed GR layers to a 3D continuous conductive network. This interconnected structure serves as an optimal transmission pathway for free electron gas, allowing for the generation of robust collective harmonic oscillations when stimulated by the incident EM field (Figure 5f). Such plasma oscillation behavior becomes particularly significant when the frequency of the EM wave falls below its characteristic frequency, resulting in a marked negative dielectric response in the material. This phenomenon also establishes a vital EM coupling mechanism for dual‐functional phase materials.^[^ ^37^ ^]^ Furthermore, infrared thermal imaging experiments (Figure 5g) demonstrated that when GR/Bi@Fe_3_O_4_ bulk samples were placed in holes of a PLA plate and subjected to continuous heating on a 60 °C platform, the temperature in the GR/Bi@Fe_3_O_4_ region increased after 100 s of thermal exposure. This observation indicates the formation of an efficient continuous thermal conduction network within the composites, significantly enhancing their thermal conductivity.^[^ ^38^ ^]^ By integrating EM simulation with thermal characterization results, this study not only elucidates the mechanism behind the negative dielectric response but also highlights the dual application potential of this material in managing EM waves and controlling thermal properties, offering valuable insights for the development of next‐generation multifunctional EM materials.^[^ ^38^, ^39^ ^]^

**Figure 6:**
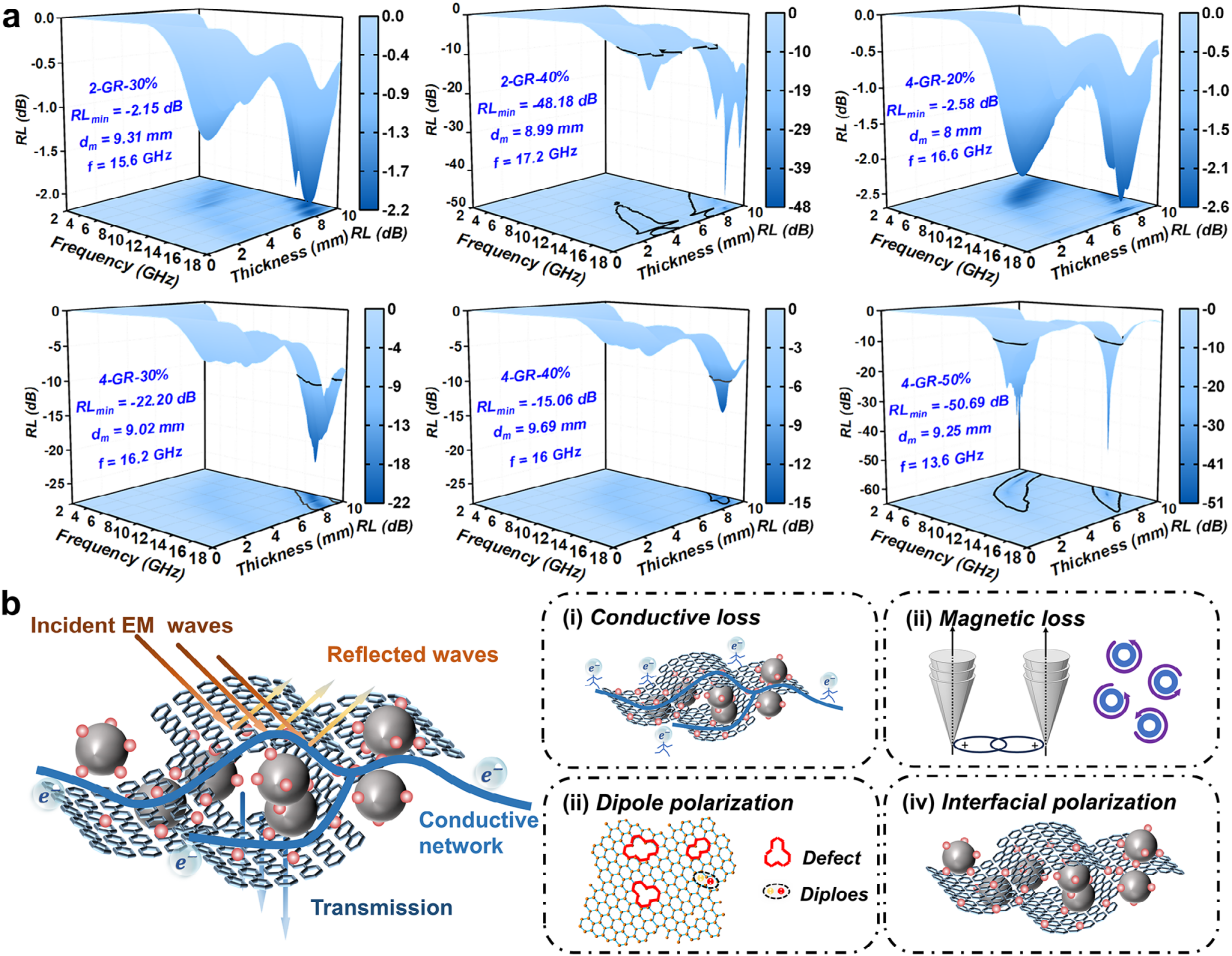
RL color maps for GR/Bi@Fe_3_O_4_ metacomposites with different fill ratios a). Diagram of the main EM wave absorption mechanism of metacomposites b).

This study investigates the potential of incorporating negative dielectric material systems into microwave absorption applications, emphasizing the unique EM response behaviors associated with this approach. As illustrated in **Figure** **6**a, we prepared samples with varying loading ratios of GR/Bi@Fe3O4 to assess their absorptive performance. The results revealed minimum reflection loss (*RL_min_*) values of −2.15 dB at 15.6 GHz for the 2−GR‐30% sample, −48.18 dB at 17.2 GHz for the 2‐GR‐40% sample, −2.58 dB at 16.6 GHz for the 4‐GR‐20% sample, −22.20 dB at 16.2 GHz for the 4‐GR‐30% sample, −15.06 dB at 16.0 GHz for the 4‐GR‐40% sample, and −50.69 dB at 13.6 GHz for the 4‐GR‐50% sample. The optimal matching thickness (*d_m_*) for achieving these *RL_min_* values is ≈9 mm. The absorptive properties of the GR/Bi@Fe_3_O_4_ composite primarily arise from interference effects linked to its structural design, coupled with the effective combination of multiple loss mechanisms^[^ ^39^ ^]^ (Figure 6b). First, the conductivity of GR facilitates the formation of an efficient conductive network. In addition, the presence of abundant interface polarization sites significantly enhances EM wave attenuation. Furthermore, the unique metallic properties of Bi form an extra electron transport pathway between GR and Fe_3_O_4_, further promoting conductive loss capability. Second, Fe_3_O_4_, as a typical ferrite material, produces magnetic loss via natural resonance and eddy current effects, while the GR network synergistically promotes eddy current effects. Moreover, the introduction of Bi effectively regulates the permittivity and magnetic permeability of the composites, optimizing impedance matching characteristics, strongly enhancing EM wave absorption efficiency. Moreover, the material's numerous structural defects act as effective polarization centers, leading to significant dipole polarization loss in the presence of an alternating EM field. The diverse heterointerfaces formed within the GR/Bi@Fe_3_O_4_ composite facilitate spatial charge accumulation due to composition gradients, resulting in pronounced interface polarization loss.^[^ ^40^ ^]^ The fundamental importance of our proposed design for dual‐functional dielectric/magnetic phase materials lies in creating a specialized EM environment that supports these interference effects.^[^ ^41^ ^]^ This framework not only provides new insights into the role of negative dielectric materials in absorption mechanisms but also challenges the conventional design paradigm that relies solely on a single loss mechanism. While there are current limitations regarding matching thickness and absorption bandwidth, this study thoroughly explores the characteristics of negative dielectric materials in microwave absorption. It fresh a novel perspective on the contribution of negative dielectric response to the absorption process and establishes a vital theoretical and experimental foundation for optimizing the performance and structural design of next‐generation EM functional materials.^[^ ^39^, ^40^, ^41^ ^]^

## 3 Conclusion

This paper introduces a groundbreaking bifunctional metacomposite, magnetic GR/Bi@Fe_3_O_4_, which achieves a tunable negative dielectric response through the creation of a 3D GR conductive network. The findings reveal that when the GR content surpasses the filtration threshold, it develops a continuous interpenetrating 3D conductive structure. In this configuration, Bi nanoparticles act as electronic transport bridges, linking GR and Fe_3_O_4_ particles. The material demonstrates a pronounced negative dielectric constant across a wide frequency range, with its frequency dependence elucidated by the Drude model, which confirms the plasma oscillation effect of free electrons within the GR network. Furthermore, the combined effects of GR's conductive loss, Fe_3_O_4_’s magnetic loss, and multiple interface polarization contribute to the material's exceptional EM wave absorption performance. Through a thorough investigation of the structure‐property relationship of the material, we have established a mechanism for regulating the negative dielectric response. This research provides valuable insights for the development of multifunctional ultra‐composite materials that integrate negative dielectric properties with EM wave absorption functionalities. This dual‐functional phase design paradigm not only expands the potential applications of ultra‐composite materials but also serves as a significant reference for the design of materials aimed at EM wave control.

## 4 Experimental Section

### Preparation

The GR sheets, with a purity exceeding 98 wt.% and dimensions ranging from 0.5 to 3 µm, were obtained from CASH Nano Technology Group Co., Ltd. High‐purity raw materials, including bismuth oxide (Bi_2_O_3_, >99%), ferric oxide (Fe_2_O_3_, >99%), and polyvinyl alcohol (PVA, >99%), were sourced from Aladdin Chemical Reagent Co. Ltd.

The synthesis process began with the preparation of bismuth ferrite (BiFeO_3_) ceramic matrix using a solid‐phase high‐temperature reaction^[^ ^42^ ^]^:

(7) $${\mathrm{Bi}}_{2}O_{3}+{\mathrm{Fe}}_{2}O_{3}\;\to \;2{\mathrm{BiFeO}}_{3}$$

Initially, Bi_2_O_3_ and Fe_2_O_3_ powders were mixed in the stoichiometric ratio, to which an additional 5 wt.% of Bi_2_O_3_ was incorporated as a sintering aid, addressing the high‐temperature volatilization of the Bi element. The mixture was subjected to ball milling for 6 h in anhydrous ethanol to ensure a uniform blend. Subsequently, the mixture was dried and passed through a 200‐mesh sieve for further refinement. The resulting powder was then placed in a muffle furnace and calcined at 850 °C for 4 h in an air atmosphere. The Bi_2_O_3_ powder obtained served as the matrix phase precursor for the composite materials.

The prepared BiFeO_3_ was finely ground and mixed with GR (2–10 wt.%) via ball milling. The homogeneous mixture was then compacted under a pressure of 50 MPa into green bodies measuring 15 mm in diameter and 2 mm in thickness, employing a 5 wt.% PVA solution as a binder. The green bodies were sintered at 950 °C for 6 h in an Ar atmosphere. During this process, BiFeO_3_ experienced thermal decomposition, resulting in the formation of Fe_3_O_4_ and the precipitation of metallic Bi on the surface of the Fe_3_O_4_ grains. The reaction can be summarized as follows:

(8) $$\mathrm{BiFe}O_{3}\underset{{\mathrm{Ar},\;950}^{\circ }C}{\to }Fe_{3}O_{4}+\mathrm{Bi}$$

The in situ‐generated metallic Bi functioned as a conductive bridge between GR and Fe_3_O_4_, significantly enhancing electrical connectivity. Finally, the sintered samples were polished to prepare them for subsequent dielectric property measurements and material characterization.

### Characterizations and Measurements

The EM and dielectric properties of the composites were systematically characterized, focusing on parameters such as complex permittivity (*ε'*, *ε''*), AC conductivity (*σ*_ac_), complex impedance (*Z'*, *Z''*), impedance modulus (*|Z|*), and phase angle (*φ*). A Precision Impedance Analyzer (Agilent E4991A, USA) equipped with a 16453A dielectric test fixture was employed for these measurements. The frequency range for testing extended from 10 MHz to 1 GHz, conducted at room temperature with a loading voltage set at 100 mV. The measurement methodology utilized the parallel‐plate capacitor method. To enhance accuracy and minimize measurement errors, a spring mechanism above the fixture ensured firm contact between the sample and electrodes, effectively reducing air gap interference. Prior to initiating formal tests, the equipment underwent open‐circuit and short‐circuit compensation, alongside load calibration, to eliminate any residual impedance.

The microstructure and chemical composition of the GR/Bi@Fe_3_O_4_ composites were comprehensively analyzed using various characterization techniques. Scanning electron microscopy (SEM, SU‐70) combined with energy dispersive spectroscopy (EDS), provided insights into the surface morphology. Transmission electron microscopy (TEM, JEOL‐1230) offered further structural details, while X‐ray diffraction (XRD, X'Pert Pro) was used to assess crystalline phases. Raman spectroscopy (LabRam HR Evolution, France) using 532 nm laser excitation contributed additional information about molecular vibrations. Simultaneous thermal analysis (STA499F3, Netzsch) integrating thermogravimetry and differential scanning calorimetry (TG‐DSC), evaluated thermal stability. Vibrating sample magnetometry (VSM, BHV‐525, Riken Denshi), measured magnetic properties, and X‐ray photoelectron spectroscopy (XPS, ESCALAB 250, Thermo Fisher Scientific) provided insight into elemental composition and chemical states.

For EM simulation, computer simulation technology (CST) was employed, with a model designed as a plate structure measuring 600 mm × 600 mm × 0.1 mm positioned on the YOZ plane. The simulation utilized a plane wave propagating in the –x direction as the excitation source, characterized by an electric field amplitude of 1 V m^−1^ and polarization along the +*y* axis. The thermal performance of the bulk sample was assessed under ambient conditions, specifically at room temperature and atmospheric pressure, using an infrared thermal camera (H21pro, Hikvision). The sample was placed in a circular cavity (Φ15 × 2 mm) on a polylactic acid (PLA) plate (95 × 60 × 4 mm) and situated on a heating platform (BY1010, BYA) maintained at 60 °C. Surface temperature distribution was meticulously recorded at 10‐s intervals over 100 s to analyze heat dissipation dynamics.

The samples containing GR at concentrations of 2 and 4 wt.% were uniformly mixed with paraffin, achieving filler loadings between 20 and 50 wt.%. These mixtures were then compacted into toroidal specimens with an inner diameter of 3.0 mm and an outer diameter of 7.0 mm for EM characterization. Using transmission line theory, the reflection loss (*RL*) was calculated with a vector network analyzer (R&S ZNB‐40) to assess the EM wave absorption capabilities of the samples. The input impedance (*Z_in_*) and the corresponding *RL* at the air‐absorber interface were determined using the following formula^[^ ^43^ ^]^:

(9) $$Z_{in}=Z_{0}=\sqrt{\frac{\mu_{r}}{\varepsilon_{r}}}\;\tanh\left(i\frac{\omega d\sqrt{\mu_{r}\varepsilon_{r}}}{c}\right)$$

(10) $$RL=20\log\left|\frac{Z_{in}+Z_{0}}{Z_{in}-Z_{0}}\right|$$

where *ω*, *μ_r_*, *ε_r_*, *d*, and *c* are the EM wave frequency, complex permeability and permittivity, sample thickness, and light velocity, respectively.

## Conflict of Interest

The authors declare no conflict of interest.

## Supporting information



Supporting Information

## Data Availability

The data that support the findings of this study are available from the corresponding author upon reasonable request.
