# Supplementary material for: Epsilon‐Negative Derived Electromagnetic Wave Absorption of Magnetic Metacomposites by Bifunctional Phase Paradigm
Source: Adv Sci (Weinh). 2025 Nov 18;13(7):e16616. doi: 10.1002/advs.202516616 (PMC12866860; doi:10.1002/advs.202516616)
Supplement: Supplementary file 1 — Supporting Information [file ADVS-13-e16616-s001.docx]

**Supplementary materials for:**

**Epsilon-Negative Derived Electromagnetic Wave Absorption of Magnetic Metacomposites by Bifunctional Phase Paradigm**

Xiance Zhu ^a^, Yunpeng Qu ^a,^ *, Qiuyun Yang ^a^, Jing Mao ^a^, Xiaosi Qi ^a,^ *, Yunlei Zhou ^b,^ *, Chunyuan Deng ^c,^ *, Shicheng Qiu ^d^, Yao Liu ^e^

^a^ College of Physics, Guizhou University, Guiyang 550025, China

^b^ Hangzhou Institute of Technology, Xidian University, Hangzhou 311231, China

^c^ School of Electronic Engineering and Automation, Guilin University of Electronic Technology, Guilin 541004, China

^d^ Department of Electronic & Computer Engineering, Hong Kong University of Science and Technology, Hong Kong 999077, China

^e^ Key Laboratory for Liquid-Solid Structural Evolution and Processing of Materials (Ministry of Education), Shandong University, Jinan 250061, China

*Corresponding authors: ypqu@gzu.edu.cn (Y. Qu), xsqi@gzu.edu.cn (X. Qi), zhouyunlei@xidian.edu.cn (Y. Zhou), dcy0606dcy@guet.edu.cn (C. Deng)


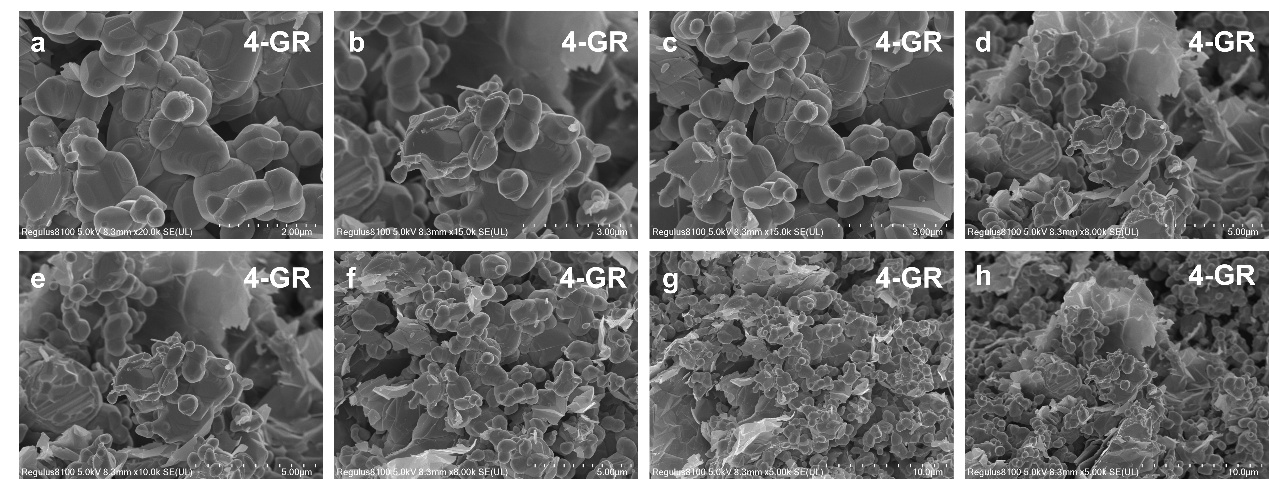

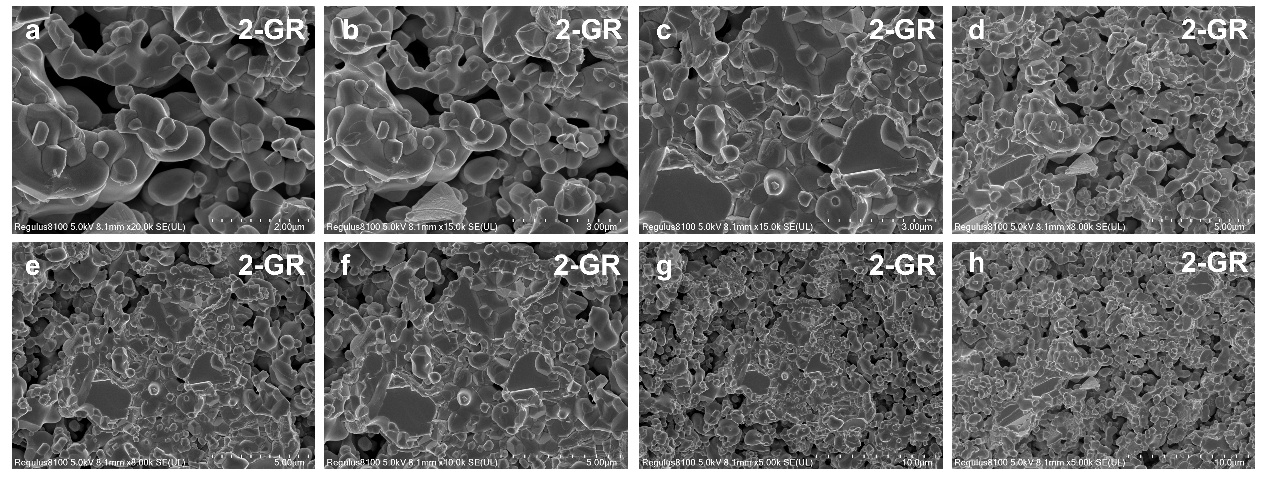
**Fig. S1.** FESEM images of 2-GR sample (a-h).


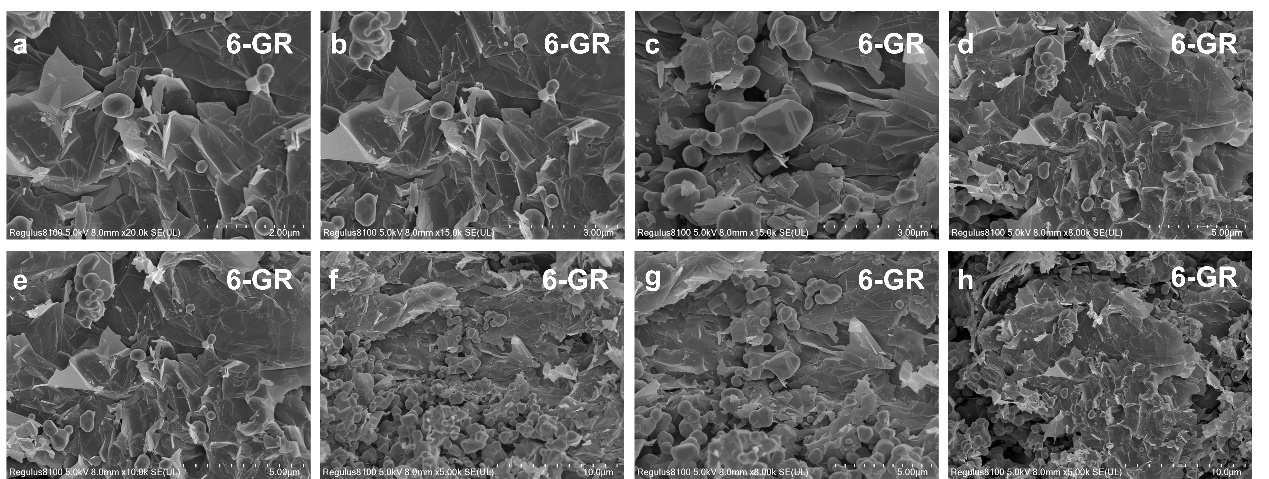
**Fig. S2.** FESEM images of 4-GR sample (a-h).

**Fig. S3.** FESEM images of 6-GR sample (a-h).


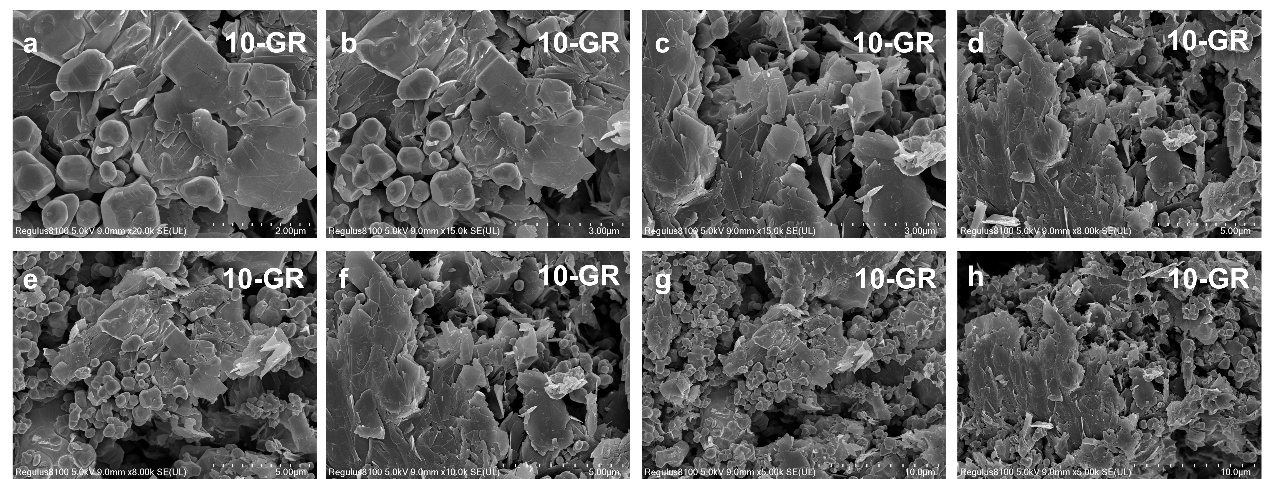

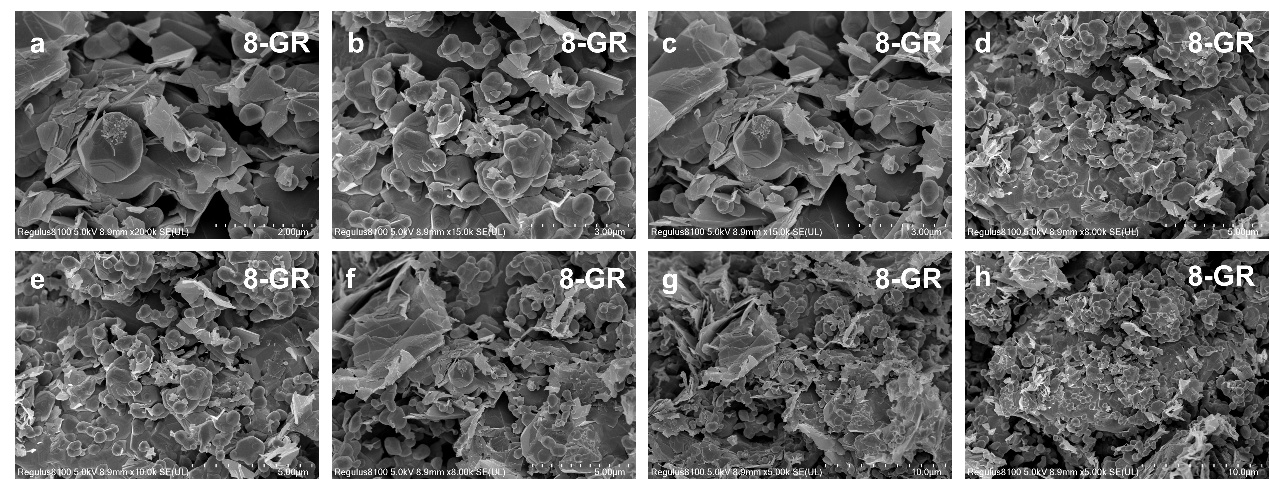
**Fig. S4.** FESEM images of 8-GR sample (a-h).

**Fig. S5.** FESEM images of 10-GR sample (a-h).


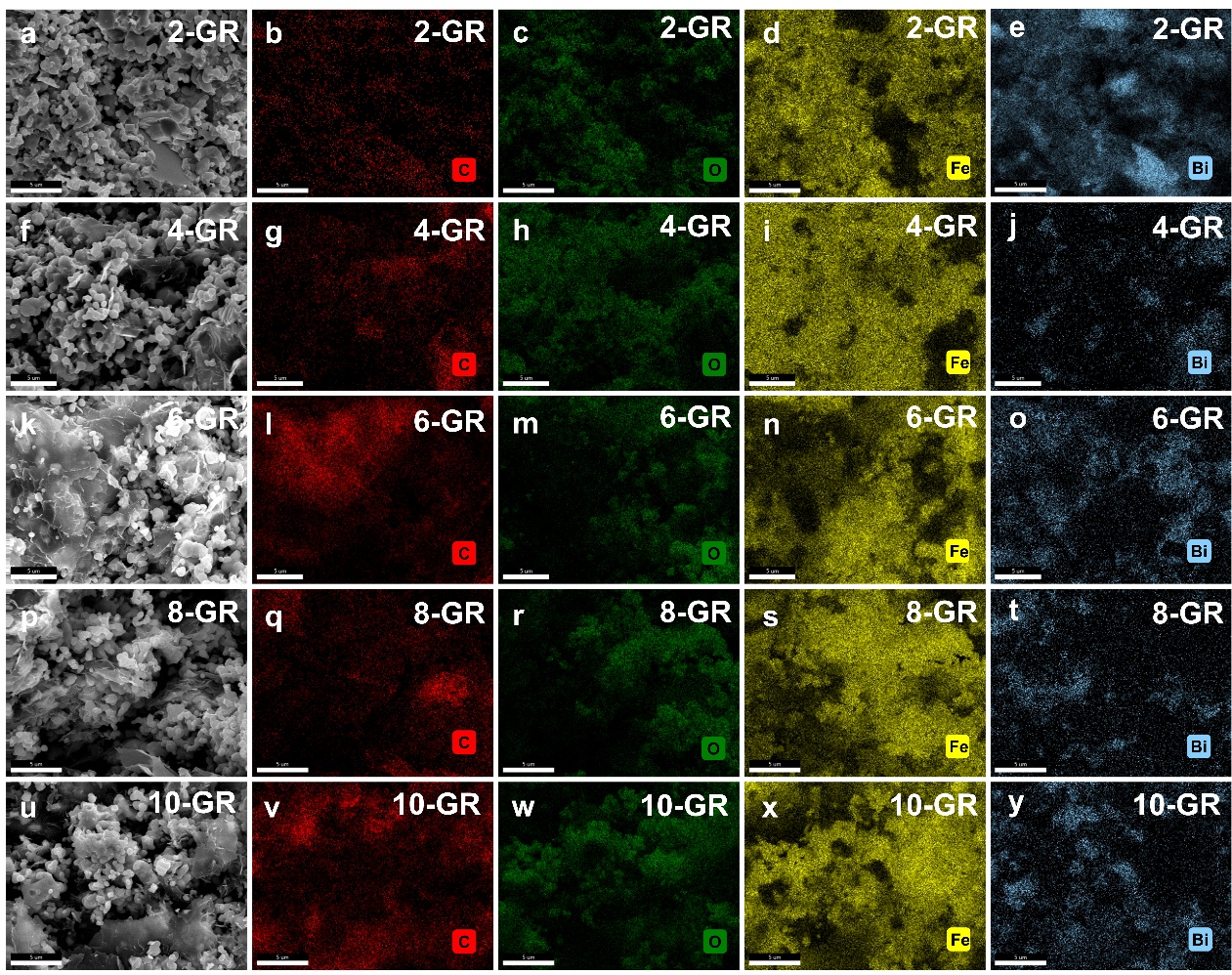

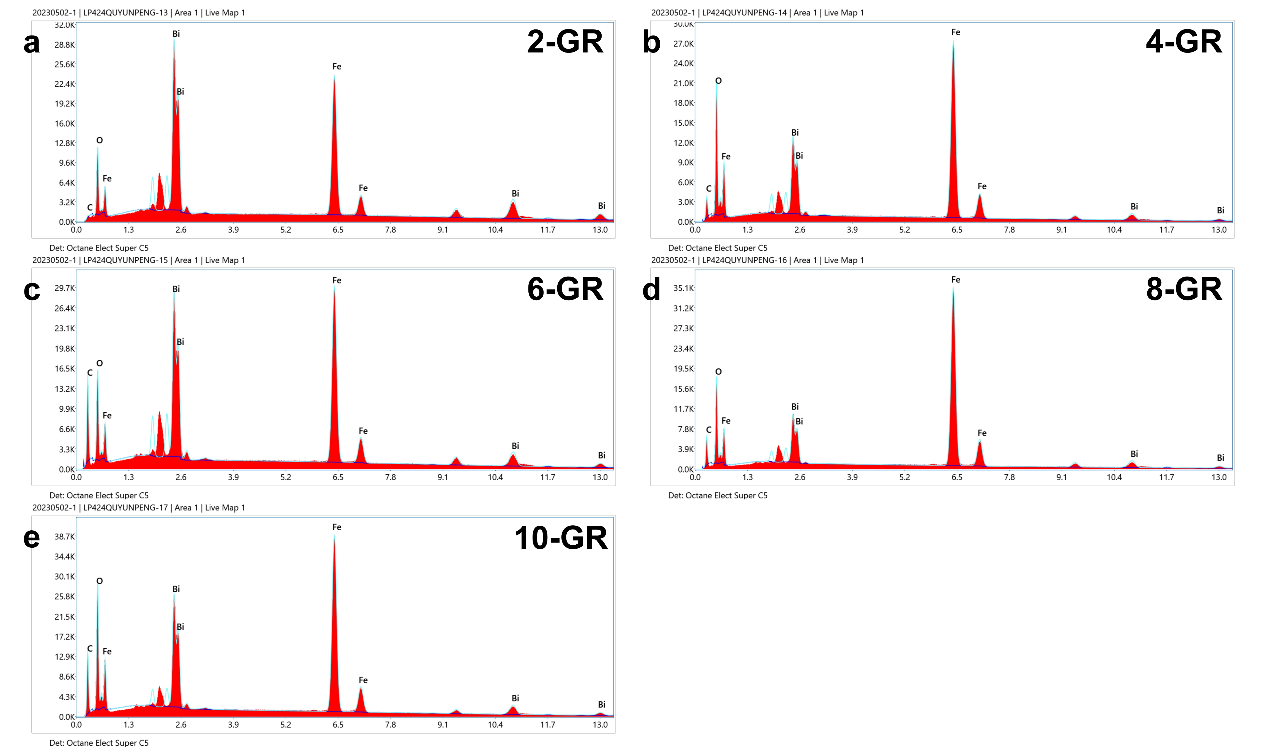
**Fig. S6.** FESEM images and EDS maps of 2-GR, 4-GR, 6-GR, 8-GR and 10-GR samples (a-y).

**Fig. S7.** Element content statistics based on EDS results for 2-GR, 4-GR, 6-GR, 8-GR and 10-GR samples (a-e).


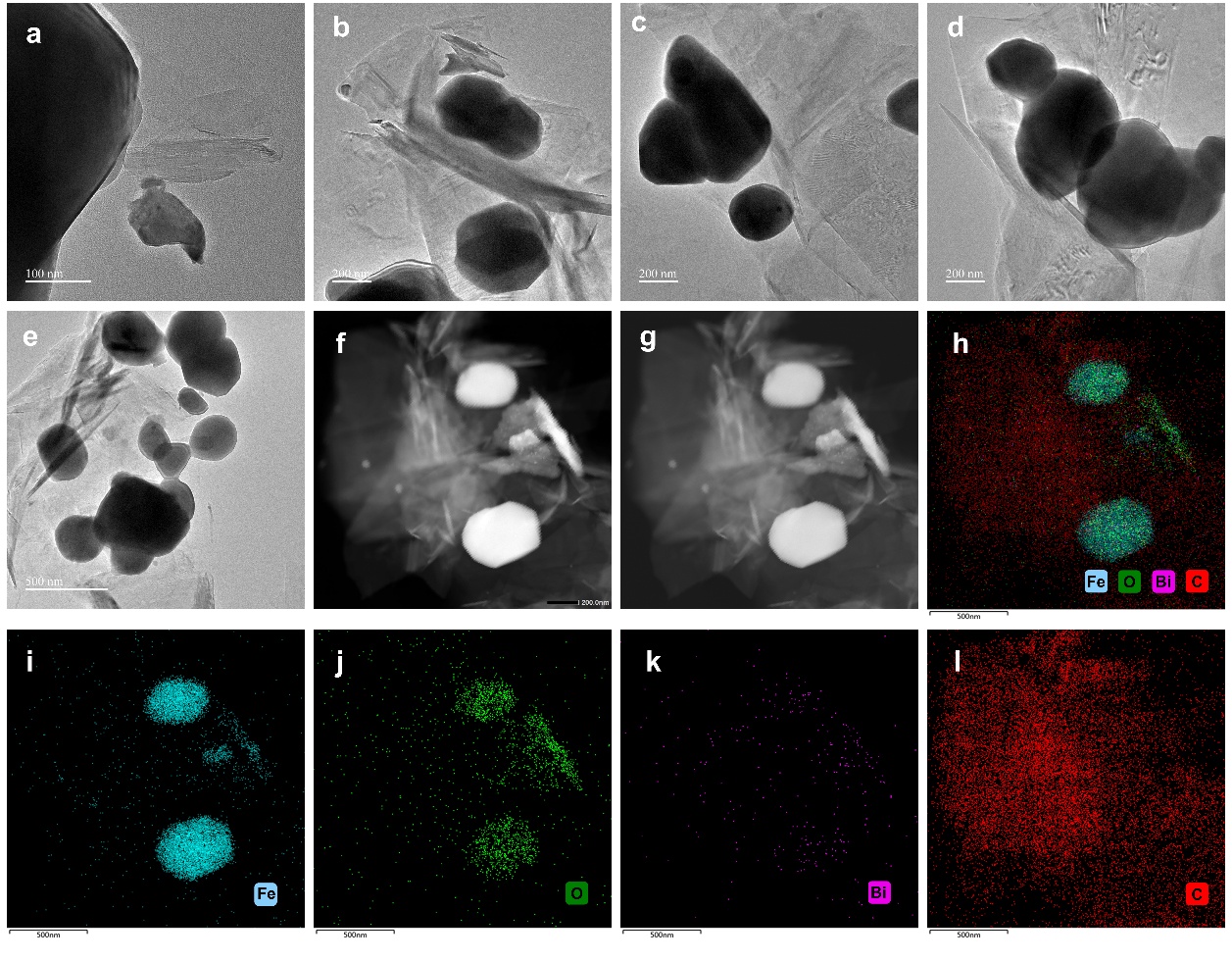


**Fig. S8.** TEM images and Mapping images of 10-GR sample (a-l).

**Fig. S9.** XRD of GR/Bi@Fe_3_O_4_ composites with different mass ratio of GR fillers.
